# Social Media Effects Regarding Eating Disorders and Body Image in Young Adolescents

**DOI:** 10.7759/cureus.58674

**Published:** 2024-04-21

**Authors:** Khushi Suhag, Shyambabu Rauniyar

**Affiliations:** 1 Medicine, Jawaharlal Nehru Medical College, Datta Meghe Institute of Higher Education and Research, Wardha, IND; 2 Anatomy, Jawaharlal Nehru Medical College, Datta Meghe Institute of Higher Education and Research, Wardha, IND

**Keywords:** consuming, body dysmorphia, twitter, social network, tiktok, instagram, social, facebook, online, internet

## Abstract

The following study investigates how eating problems and young people's body image are affected by social media. As social media becomes more pervasive, young teenagers are exposed to many pictures, messages, and influencers encouraging unachievable body and beauty ideals. The study investigates the link between social media usage and the development of binge eating disorder patterns and undesirable body image judgments. It also sheds light on the influence that so-called social media influencers and celebrities have on encouraging disordered eating patterns and forming negative body image perceptions. On websites like Instagram and TikTok, exposure to deliberately selected photographs of "ideal" body types encourages body dissatisfaction and poor self-worth. The research emphasizes social media's possible harm to this vulnerable group's physical and mental health by examining pertinent literature and empirical evidence. It also looks at potential safeguards and methods for fostering good mental and physical health in early adolescents during pervasive social networking site use. Promoting fad diets, weight-loss aids, and the exaltation of extreme thinness aids in the normalization of harmful behaviours. Social media's negative consequences on eating disorders and body image in young teenagers are highlighted by this study in its conclusion. As a critical developmental stage, early adolescence is vulnerable due to the effect of social media, which could normalize unhealthy behaviours and maintain negative body image and insecurities. The findings underscore the need for targeted interventions and educational programs to mitigate the adverse effects of eating problems and young people's body image in light of social media.

## Introduction and background

Teenager's usage of social media and the Internet is snowballing, making eating disorders more likely to develop and persist by introducing new dynamics and risks [1]. While eating disorders were once predominantly associated with Western societies, the emergence of reports detailing cases of eating disorders in non-Western cultures has expanded the perception of these disorders as a global issue [2]. The prevalence of eating disorders among adolescents has garnered increasing attention in recent years, coinciding with the rise in social media usage among this demographic. Adolescents, particularly those in the age range of 13-18, are among the heaviest users of social media platforms, with platforms like Instagram, TikTok, and Snapchat serving as prominent avenues for social interaction and self-expression.

It is becoming more popular to research the psychological impact of utilizing and using social networking sites (SNS). SNS utilization has been connected to both good and bad outcomes so far. SNS usage has been connected to increased loneliness and enhanced social connectedness and well-being. The term "body image" refers to an individual's opinions, sentiments, and ideas on their looks. When a person has negative body perceptions and perceives a disconnect between their actual and ideal bodies, it is said that they are experiencing body dissatisfaction. This shift in awareness can be attributed, in part, to the influence of mass media in shaping body image perceptions. Traditional mass media has traditionally portrayed the "ideal" body type, incorporating television and publications for women's fashion. According to research, these images generally showcase gorgeous, heavily photo-shopped ladies who are frequently young and tall, with modest breasts, and who are incredibly slim. Numerous correlational and experimental research studies have been conducted to support the connection between media exposure, food addictions, and body dissatisfaction in young people, especially females. Among the most commonly accepted theories that have been tried to explain how media operates and influences diet and perceptions of one's physique are the sociocultural and objectification theories [3].

Despite the ongoing widespread use of conventional media, other "new" media, most notably the Internet, are being utilized increasingly often. According to the Internet Growth Statistics (2014), the amount of time spent online has increased by almost 50% per year since 1990; according to estimates from July 2014, 40% of the world's population used the Internet. Additionally, undergraduate women prefer image-focused websites over fashion publications at the moment. Examples of these websites include fashion/beauty and entertainment/celebrity websites. According to a recent study, many websites promote stereotypical notions of feminine elegance, just like in the mainstream media. For instance, a study was conducted by Slater, Tiggemann, Hawkins, and Werchon (2011) on content analysis of commercials on websites targeted at teenagers and discovered that most of the figures in the advertisements were female, young, slender, and pretty. According to a study of high school females, Internet use was also connected to increased internalization of the slim ideal, comparison of appearances, unhappiness with one's weight, and a desire for thinness. Similarly, it was discovered that young ladies who utilize the Internet have a higher risk of developing eating problems [3]. Body image research goes past the thin ideal, even if being unhappy with one's physique can be connected to a range of body components (such as skin tone, eye shape, and breast size) [4].

Everywhere, social media use is pervasive and growing swiftly. The most popular social networking platform is Facebook, which has over 1.3 billion regular users. Compared to males, young women are more likely to use social media, and this group also experiences body dissatisfaction more frequently than boys. The social comparison theory contends that people must evaluate their development and standing in various spheres. People evaluate their standing by comparing themselves to others without objective standards, and a greater predisposition to do so is associated with significant body dissatisfaction. Most experimental research says that the impact of social comparisons based on looks and body image focused on how body dissatisfaction affects young females is impacted by exposure to idealized bodies in traditional media like publications, broadcast media, and music videos. The results of this study show that women and girls exposed to the slender ideal have more negative body images. More recent surveys indicate that the attractiveness of traditional media types, particularly among teens, is diminishing due to increased accessibility to and popularity of interactive media, including the Internet. Exposure to optimal thin media, such as magazines, affects body dissatisfaction and mood [5].

Teenage females are at an alarmingly high risk of developing eating problems, which have been linked to severe emotional anguish and medical issues. Compared to boys, girls are three times more likely to exhibit an eating disorder by the age of 15 years. Literature reveals that due to the developmental changes brought on by puberty, as well as cultural and societal pressures, body image is a problem for people throughout their entire lifespan. However, it is most prevalent in adolescents [6]. The American Psychiatric Association claims that subclinical levels of having an eating disorder affect up to 10% of adolescent females. Equal to adult populations, adolescents also experience anorexia nervosa and bulimia nervosa at similar rates. As a result, researchers have worked extremely hard over the past few years to identify, quantify, and assess the formative factors that can trigger the start of such disruptions [7].

Researchers have worked incredibly hard over the past few years to discover, quantify, and evaluate the formative elements that can start such disruptions. It is feasible to hypothesize an increase in eating disorders and body image problems because of this overemphasis on physical attractiveness online possibly even more so than offline. The presence of Internet communities like Reddit and Quora is, last but not least, supporting radical methods of weight loss and management, such as websites and Internet resources that promote eating disorders and perhaps encourage the formation or maintenance of eating disorders and problems with body image. In conclusion, there are a variety of ways that social media and Internet use may affect problems with food and body image [8]. It is imperative to conduct extensive research on the link between eating disorders and social media to fully grasp the intricate relationship between media exposure, body image perceptions, and mental health. This research is critical in developing evidence-based strategies to promote healthy behaviours and mitigate the harmful effects of social media on vulnerable populations.

Additionally, several circumstances make discussing the connection between the Internet and problems with food and one's body particularly important among teens and newly minted adults. This is the most active age group in terms of social media use. Second, many significant developmental difficulties during adolescence may be found on the Internet, notably regarding social development and issues like identity, self-worth, peer connections, and health behaviours that are also crucial for food and body image issues. In this way, it has been demonstrated that SNSs and the Internet significantly affect teenage relationships as a crucial setting for adolescent identity formation. According to particular views, peers may also influence a person's personality traits, physical characteristics, and behavioural tendencies, particularly throughout adolescence [9]. Additionally, in line with the features of the Internet that were mentioned above that are pertinent and devoted to the research concerning eating disorders and a person's body image, the Internet has been conceptualized within developmental psychology as a type of media, both a cultural tool and a means of communication. An excellent theoretical base supports a connection between worries about food and juvenile body image and the Internet and social media use [8].

## Review

Search methodology

**Figure 1:**
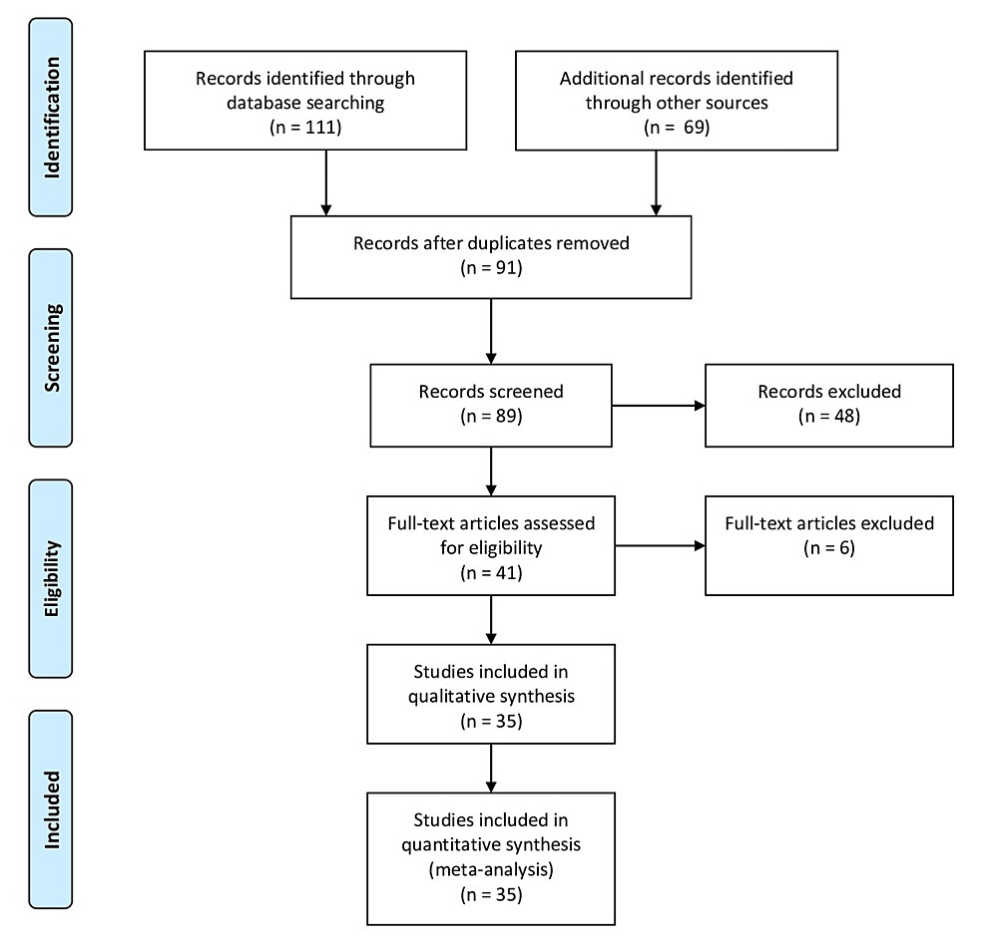
PRISMA flow diagram for screening and selecting articles related to social media effects regarding eating disorders and body image PRISMA: Preferred Reporting Items for Systematic Reviews and Meta-Analyses

Electronic databases like PubMed and Google Scholar, books, and grey literature were searched and used to find relevant studies. The search was not time-limited because it was assumed that most research would be current. The articles' reference lists were also searched for further possibly pertinent articles. The words "Internet," "Online," "Facebook," "Social," "Instagram," "Tik-Tok," "Social Network," "Snapchat," "online platforms," "internet communities," "online forums," and "Twitter" were used as key phrases. The terms "Body Dysmorphia," "Disordered eating," "Self-objectification," "Self- Surveillance," and "Social Comparison," "Binge Eating," "Binge Eating Disorder," "Social identity," "Anorexia," "Bulimia," and "Body Image" were also used. Boolean operators (AND, OR, NOT) were utilized to combine the search terms effectively and refine the search results. Epidemiology, links, effects, health problems, and treatments were all included in the literature review. The Preferred Reporting Items for Systematic Reviews and Meta-Analyses (PRISMA) flow diagram depicts the search methodology of the review article given in Figure 1.

Epidemiology

Eating disorders are expensive, fatal, and debilitating mental conditions that severely impair psychosocial functioning and physical health. Disturbed beliefs about eating disorders can result from thoughts about food, weight, and body image. In the previous 50 years, there has been a correlation between variations in the food environment and a rise in eating disorders. Every healthcare provider should routinely inquire about the patients' eating routines to evaluate their general health. Anorexia nervosa, disorders of binge eating, avoidant-restrictive food intake, bulimia nervosa, pica, and rumination disorder are among the feeding and eating disorders that are currently recognized in diagnostic systems. For instance, eating disorders may manifest differently in men and women because there is not enough research and analysis on eating disorders; there is a lot of uncertainty about their origins, therapy, and management [10].

Nearly all eating disorders involve a focus on weight, body image, and eating that is excessive, which could lead to dangerous eating patterns. These measures may significantly harm the body's ability to absorb nutrients. Eating disorders can injure the mouth, bones, teeth, heart, and digestive system. They might cause other diseases. They have also been linked to self-harm, anxiety, suicidal thoughts and actions, depression, and feeling blue. With the proper care, one can re-establish healthy eating patterns and cultivate healthier perspectives on food and the body. The person may also be able to mitigate or reverse the severe consequences that the eating problem has caused. What precisely causes eating disorders is uncertain. There may be several reasons, similar to other mental diseases. First is genetics: Some people's more significant risk of having eating problems may be influenced by genes. Second is biology: Changes in brain chemistry and other biological variables, such as genetics, may have an impact on eating disorders [11].

Body dysmorphic disorder (BDD), which manifests as a stressful or crippling fixation on an alleged or inconsequential physical fault, typically manifests throughout adolescence. However, the clinical characteristics of BDD in this age group (adolescents) have received microscopic study. BDD is a disorder that is frequently very severe and that disrupts typical teenage development. Additionally, adolescents must be confident in their favorable body image, which is related to sadness and anxiety. It might be the main element impacting teens' general self-esteem and the fear of being poorly assessed by people in their age group [12].

In contrast to earlier Diagnostic and Statistical Manual of Mental Disorders (DSM) versions, which put BDD in the category of somatoform disorders, BDD is now classified in DSM-5 as the group of illnesses known as obsessive-compulsive disorder (OCD) and related disorders. This change in how BDD is thought of aligns with scientific research, which demonstrates that BDD and OCD are separate but closely linked mental diseases that are frequently co-occurring [13]. BDD is a separate comorbid illness that affects nearly half of eating disorder patients [14], and body image issues are closely correlated with BDD and a dangerous element for the development and maintenance of eating disorders [15].

The link between social media, eating disorder, and body dysmorphia

The sociocultural media's impact on how people feel about their bodies and disordered consumption has been the subject of research for more than 20 years [16]. It has been recognized that the media can affect people with eating disorders. Compared to traditional media (like television), self-objectification characteristics may be more strongly influenced by Internet media. The usage of various SNSs as sources of knowledge and inspiration for notions of health and beauty has expanded. Users might be tempted to evaluate their physical similarities to unattainable and unreasonable physical standards on these platforms. SNS use has been linked to concerns about how eating disorders may develop or persist. Recent SNS trends, like "fitspiration," which combines the words "fitness" and "inspiration," idealize intense pictures of people with incredibly lean bodies.

In contrast, other SNS trends like "thinspiration" or "bone-spiration" highlight physical changes due to particular eating disordered behaviours, like restricting one's diet and extreme weight loss. Two years later, a prospective relationship between SNS usage and a rise in the urge due to thinness was discovered, indicating a possible causative relationship between social media use and eating disorder cognitions. Studies on specific groups have revealed that they struggle with inhibitory and executive control (impulse control), comparable to individuals battling addictive behaviours [17].

Typical portrayals of beauty standards may be seen on several social media platforms. SNS development and growth, including that of Facebook and Instagram, contribute to the spread of the thin-and-fit ideal. Additionally, because social media is a tool that teenagers frequently use associated with worries about individual liberty, monitoring one's behaviour, having obsessions, and using social media compulsively, it is a factor that allows attention to one's appearance. It is also evident from having access to social media features like facial filters and photo editing tools [18]. Personalized material, frequently less moderated, more intense, and intended to keep users interested for longer durations, is made available to viewers through algorithms, intensifying this relationship [19]. The lockdown has also impacted SNS usage, which may be connected to a rise in eating disorders and the desire for thinness among teenagers and young women [20].

Some people object to bodily changes from a network because of the media's criteria for beauty. Although the physical changes that adolescents go through are common, some people nevertheless do not accept these changes. Humans undergo physical changes during middle age, including the skin that shrinks and becomes less firm, thins, and turns white, together with changes in body weight. The person's perception of their body is always tied to physical changes. In implementing a beautiful concept, one declares that one's interpretation of beauty will impact how people perceive and uphold normative standards. As a result, most middle-aged adults and adolescents pay excessive attention to physical body alterations and strive to maintain a desirable physique. According to study findings, BDD tends to more frequently impact young individuals between the ages of 15 and 30 [18]. Spending more time online, especially in self-image- and eating-related activities, was associated with less satisfaction with one's body image and unhealthy eating habits [21].

Effects on adolescents

Self-esteem is a highly effective predictor of various human behavioural variables, including health and well-being. According to several studies, women, in particular, are thought to find that self-perception and self-esteem, overall mental health, and eating behaviour are all strongly correlated with one another. In this way, having high self-esteem aids young people in making better psychological adjustments, facilitating their socialization and serving as a protective factor against risky behaviour. As a mediating factor for eating disorders, self-esteem may be linked to how one constructs their body image, particularly throughout adolescence. Low self-esteem frequently coexists with the psychopathologies of eating disorders because eating disorder symptoms worsen low self-esteem, creating a vicious cycle of maintenance [22].

While interest and worry regarding the influence of SNS usage on bodily perception and the psychopathologies of eating disorders are growing, the collection of knowledge regarding how eating disorders and SNSs interact and have an impact on people is constantly expanding. Mental health specialists have been highlighting problematic practices in this area, such as excessive time spent on social media platforms or posting images that encourage attitudes and actions related to disordered eating. Additionally, based on the media exposure to SNSs, there are significant pressures to lose weight through the promotion of thinness and the visualization of diet-culture material, as well as exposure to commercials, advertising, and food-related marketing; this together might result in risk factors including body dissatisfaction, use of diets, or bulimic behaviours. In addition to social media exposure, the dynamics of involvement through "likes" and comments on the posted photographs are also crucial for understanding eating disorders [22].

Several ladies are within the normal weight range and experience unhappiness with a characteristic of their weight or body type despite rising obesity rates in the broader population and the healthiness of various weight control strategies. The term "normative discontent" has been used to characterize how ubiquitous body image issues are among women in our society. Currently, prepubescent girls are also affected by this obsession with body image, which previously only affected adult and adolescent women [23]. Adolescent girls feel poorly about their body size or shape in 50-88% [24]. According to specific theories, a person's peers may also affect their personality traits, physical attributes, and behavioural tendencies, especially throughout adolescence [25].

Men have received less research attention than women on how SNS and problems with food and body image are related. It is more probable for boys and men to utilize SNS like Twitter and YouTube that offer information. However, they also engage in behaviours focused on appearance, such as editing and publishing pictures on Instagram, frequently in the form of "selfies." Additionally, they might look for material about muscles and use SNS practices associated with this obsession. The highest risk for muscularity concerns and disordered eating appears to be conferred by appearance-based SNS behaviours. People who seek out SNS content about nutrition and muscularity and participate when compared socially to other users in their network may be particularly at risk [26].

Body image and disordered eating behaviours as health problems

There are associations between negative body image and other aspects of wellness and mental health, according to numerous research. The detrimental impact of body image on society dissatisfaction can include diminished sexual function, avoidance of social situations, and poor self-esteem. These conditions can include depression, anxiety, and BDD. One may radically modify their eating habits to transform their look and achieve the slender frame portrayed in Western media if they are unhappy with their weight or shape. Additionally, a key risk factor for starting disordered eating is having a negative body image. A concern when weighed or form is a common symptom of eating disorder patients, including an obsession with body fat deposits, a steadfast drive to lose weight, and a great fear of gaining weight. The symptoms of an eating disorder can also include a variety of additional thoughts and emotions, such as obsession with a meal's calorie content, frequent food-related thoughts, discomfort after eating desserts, and guilt after eating. Behaviour characteristics of an eating disorder include a significant reduction in caloric intake, bingeing episodes followed by purging (self-induced vomiting, use of laxatives), slicing food into tiny pieces, eating slowly, and avoiding meals heavy in sugar or carbs. These kinds of behaviours can have detrimental effects on one's health. The mortality rate of eating disorders, specifically the worst mental illness, is anorexia nervosa, second only to opioid addiction. Cardiac arrhythmia, amenorrhea, bradycardia, weakening of the heart muscle, esophagitis, and esophageal or gastric rupture are just a few of the medical complications that can result from eating disorders. Enduring eating disorder patients may experience lasting repercussions, including weakening of bones and a higher risk of premature birth, bleeding and nausea during labour, and miscarriage, even with medical treatment [27].

Eating disorders and their sequelae

Eating problems affect 5% of female teenagers and young adults. Anorexia nervosa is connected to several medical conditions, including hypothalamic amenorrhea, whereas bulimia nervosa can be challenging to treat, especially if there is a history of sexual abuse. Instead of using sex hormones as a supplement, establishing healthy eating habits should be the primary treatment for amenorrhea [28].

Adolescents frequently have eating disorders, which considerably negatively impact their physical and mental health. The most common justification for seeing a gynaecologist is amenorrhea, one of the hallmarks of anorexia nervosa. The occurrence of amenorrhea in a young lady should make the gynaecologist suspect an underlying eating disorder. Long-term amenorrhea with a low estrogen status can result in the potentially irreversible consequence of osteopenia. The best way to treat eating disorders is through a multidisciplinary team effort that includes a doctor, dietitian, and therapist. The gynaecologist should abstain from only providing estrogen replacement therapy without additionally sending a patient for in-depth treatment or therapy of the eating disorder as it has not been demonstrated to be an effective treatment for osteopenia in anorexia nervosa. The cornerstones of medical management are nutritional rehabilitation, weight restoration, and the return of natural menstruation. Doctors should advise taking calcium and vitamin D supplements and engaging in moderate weight-bearing activity where necessary. Alendronate, dehydroepiandrosterone (DHEA), and insulin-like growth factor-1 (IGF-1) are among the more recent medicinal alternatives for the treatment of osteopenia [29].

What can be done to prevent eating disorder and its treatment?

The development of eating disorders in teenagers is significantly influenced by social pressure and the fixation on thinness [30]. The initial sign of an eating disorder's onset appears to be inadequate nutritional intake, followed by the employment of incorrect weight-loss techniques and an excessive increase in exercise time and load [31]. Teenage body shaming and bullying are two significant factors in the over-focus on weight and appearance, the development of negative self-perceptions, and unhappiness with specific body features [32].

So, what we can do to help people with eating disorders is to encourage their families to encourage healthy eating and exercise more at home and refrain from discussing weight issues. Families should be encouraged to have more frequent and wholesome family dinners, discourage excessive diets, and encourage a positive body image. Consider that obese teenagers have been subjected to weight discrimination, and talk to the teens about this issue. These suggestions emphasize the significance of assisting adolescents and their families to lessen the emphasis on weight and more on permanent behavioural change [33].

There are several warning signs of an eating issue, some of which are displayed in Table 1.

**Table 1:** Several warning signs of an eating issue. Image Credit: Self-created

Sr. no.	Several warning signs of an eating issue
1	Making excuses to avoid eating or having a snack
2	Without a doctor's prescription, consuming an incredibly limited diet
3	Placing an excessive amount of focus on nutrition or good eating, especially if it prevents one from taking part in customary events such as birthday cake, sporting banquets, or dining outside
4	Making your own food instead of consuming what your family does
5	Absence from customary social activities
6	Constant worry or complaints about being overweight or unwell, along with suggestions for diet plans
7	Constantly examining oneself in the mirror to look for perceived imperfections
8	Constantly consuming a lot of food
9	Using herbal remedies, laxatives, or dietary supplements to lose weight
10	Working out far more than the ordinary person. This includes skipping rest days or days off due to an illness or injury, as well as declining to go to social gatherings or other important life events because you want to exercise
11	Knuckle calluses from sticking fingers in mouths to make people throw up
12	Enamel loss issues that could be a sign of frequent vomiting
13	Leaving to use the toilet during or immediately following meals
14	Discuss despair, abhorrence, humiliation, or guilt related to eating behaviours
15	Eating covertly

Physicians, mostly pediatricians, must consider these illnesses in the proper therapeutic situations, assess individuals who may have these problems, and manage (or refer) patients who have been diagnosed with eating disorders, as recent years have seen a dramatic increase in the incidence and prevalence of eating disorders in children and adolescents. Pediatricians are expected to support laws and regulations that guarantee adequate services, such as medical attention, nutritional counseling, psychological treatment, and care coordination for those with eating problems [34].

Children and teenagers with bulimia nervosa and anorexia nervosa present differently than they do in grownups. Diagnostic procedures and multidisciplinary therapies must be specific to meet the unique developmental, nutritional, medical, and psychological needs of children and adolescents with eating disorders [35].

Many people with eating disorders don't think they need therapy. One of the main traits of many eating disorders is the inability to recognize how severe the symptoms are. Guilt and shame are further deterrents to obtaining assistance. Encourage a friend or relative struggling with an eating issue to get medical help. Even if the other person isn't ready to admit an eating disorder, one might start the dialogue by showing care and wanting to listen [11].

Risk factors and prevention

Anyone can develop an eating disorder. Although eating disorders can happen regardless of age, they frequently begin in the teen and young adult years [11]. Several factors, including those listed below (Table 2), may have an impact on the chance of developing an eating problem.

**Table 2:** Factors that may have an impact on the chance of developing an eating problem. Image Credit: Self-created

Sr. no.	Factors that may have an impact on the chance of developing an eating problem
1	A family tree: Individuals are more prone to develop eating disorders if eating disorders were experienced by their parents or siblings.
2	Additional mental health problems: Depression, anxiety, trauma, obsessive-compulsive disorder, and mental health issues may raise the risk of developing an eating disorder.
3	Diets and starvation: Weight fluctuations brought on by constantly starting and stopping different diets raise the risk of developing an eating disorder. Several eating problem symptoms include indicators of starvation, based on convincing evidence. Hunger-related brain changes include anxiety, mood swings, rigid thinking, and decreased appetite.
4	A pattern of bullying related to weight: Bullying and taunting about weight are two situations that may lead to the likelihood of someone acquiring eating problems. This applies to anyone who has faced and experienced stigma connected to their weight from peers, healthcare providers, coaches, teachers, or family members.
5	Anxiety: Adaptation may be stressful, if it is a new college environment, getting a new job, moving, or dealing with a problem in your family or relationships. Additionally, stress may make eating disorders more likely.

A person suffering from an eating disorder can take precautionary measures to establish good eating habits, but there is no surefire solution to stop eating disorders. *Adults*

Pick a balanced diet with plenty of whole grains, fruits, and vegetables. Limit your intake of alcohol, trans fats, saturated fats, salt, and sugar. Don't go on a strict diet. If you need to lose weight, consult a dietician or doctor to develop a strategy that works for you. Using herbal remedies, laxatives, or dietary supplements is not advised for weight loss. Spend enough time moving and exercising. Get 150 minutes or more of cardiovascular exercise per week, such as brisk walking. Pick enjoyable activities, such as swimming, so you'll be more likely to participate in them. For mental health problems, including depression, anxiety, or problems with self-esteem and body image, get help by consultation [11]. *Children*

Steer clear of diets near your youngster. Family mealtime customs may have an impact on how kids interact with food. You can talk to your youngster about the dangers of dieting while you eat meals together. It also lets you determine whether your child is consuming enough quantity and variety of meals. Interact with your kid. Many websites and other social media platforms spread harmful views, such as the idea that anorexia is a lifestyle choice instead of an eating disorder. Some websites entice teenagers to begin dieting. It's crucial to dispel false assumptions like these. Discuss the dangers of choosing unhealthy foods with your child. Regardless of your child's size or form, encourage and reinforce a positive body image in them. Discuss self-image with your child and reassure them that many body types are possible. In front of your youngster, refrain from criticizing your physique. Positive self-esteem-boosting messages can be cultivated. They can also help you develop resilience, the capacity to bounce swiftly from adversity. These abilities can aid kids in navigating the brutal teen and young adult years. Speak with your child's doctor for assistance. Healthcare professionals may be able to spot the first indications of an eating issue at well-child checks. They can enquire about the food habits of the kids. These visits may involve measurements of body mass index, height, and weight percentiles, which can notify you and your child's provider of any significant changes [11].

Table 3 depicts the summary of all the articles included in the review.

**Table 3:** Summary of all the articles included in this review. Image Credit: Self-created BDD: body dysmorphic disorder; SMAs: sexual minority adolescents; HEW: heterosexual women; HOW: homosexual women; PUI: person under investigation; SNS: social networking sites; BSQ: band sequential; BAS: Body Appreciation Scale

Author	Year	Findings
Saul and Rodgers [1]	2018	The Internet not only carries extreme information that encourages potentially hazardous eating disorder behaviours but also has a high degree of aesthetic pressure.
Chisuwa and O'Dea [2]	2010	Although eating disorders were once thought to be exclusive to Western civilization, there are claims that eating disorders are becoming increasingly common in non-Western nations like Japan.
Holland and Tiggemann [3]	2016	SNS use is linked to disordered eating patterns and negative body image. Particularly harmful SNS actions were found to include uploading and viewing images and using status updates to solicit unfavorable criticism.
Prieler and Choi [4]	2014	Body image and the influence of culture; a theoretical exposition founded on the concept of self-construal.
Fardouly et al. [5]	2015	The impact of Facebook use on women's emotions and perceptions of their bodies, how these effects vary from those of an online fashion magazine, and if the desire to compare one's looks to others mitigates any of these impacts.
Singh et al. [6]	2016	The percentage of teenagers who worry about their bodies and who are aware of the connection between media, eating habits, and well-known individuals and body image issues.
Keery et al. [7]	2004	Analysis and contrast of the partially mediated association between peer pressure and body dissatisfaction and the completely mediated relationship between parental influence and body dissatisfaction.
Rodgers and Melioli [8]	2016	The existence of material on social media and the Internet that is connected to beauty, especially material that promotes extreme body types or habits.
Franchina and Coco [9]	2018	The significance of idealized social media images in influencing teenagers' conceptions of their bodies, particularly the slender ideals for ladies and the muscular ideals for boys.
Treasure et al. [10]	2020	Eating disorders are expensive and fatal and severely harm mental health. They also interfere with psychosocial functioning and significantly worsen physical health.
Phillips et al. [12]	2006	Due to BDD, adolescents endure significant levels of anguish, and many have had suicidal thoughts or attempted suicide.
Angelakis et al. [13]	2016	Suicidality in BDD is a serious issue that hasn't gotten enough study attention. Compared to people without BDD, those with a diagnosis of BDD were twice as likely to attempt suicide and four times more likely to have suicidal thoughts.
McGuire et al. [14]	2023	Among cisgender SMAs, protective school policies and treatment practices that target internalized homonegativity may lower the incidence and prevalence of likely BDD.
Henn et al. [15]	2019	There is preliminary evidence that the way that women view their bodies varies according to their sexual orientation: HEW seem to exhibit more symptoms of body image disorder than HOW.
Harrison and Hefner [16]	2008	Eating disorders such as anorexia nervosa and bulimia nervosa are distinguished by the internalization of the thin ideal, a strong desire to be thin, and a profound dread of being overweight.
Ioannidis et al. [17]	2021	Dissatisfaction with one's physique and eating disorders are linked to PUI.
Susanti and Eriyani [18]	2022	An increased risk of body dysmorphic tendencies is associated with more frequent and severe Instagram use.
Harriger et al. [19]	2022	The literature has long established a link between body image and social media use; however, the use of algorithms by social media companies may amplify this association, as viewers are presented with personalized content that is frequently more extreme, less regulated, and intended to hold their attention for extended periods of time.
Vall-Roqué et al. [20]	2021	The number of women following appearance-focused Instagram profiles, as well as the frequency with which all analyzed social media platforms (Twitter, Facebook, YouTube, Instagram, and TikTok) were used during lockdown, increased significantly.
Frieiro et al. [22]	2022	The creation of successful therapies along these lines may prove beneficial in treating the attitudes and behaviours associated with eating disorders.
Blowers et al. [23]	2003	Social comparison also had a role in the association between internalizing the slender ideal and body dissatisfaction. It was shown that body dissatisfaction was directly correlated with body mass.
Hutchinson and Rapee [25]	2007	The impact of early adolescence's peer environment on eating disorders and body image.
Lonergan et al. [26]	2021	Research on the connection between SNS and eating disorders and body image issues in males is scarcer than in women.
Bavikatty [27]	2022	Viewing content that offers diet advice and BSQ showed unfavorable connections, according to correlation analysis. Furthermore, BAS and viewing style material showed a strong favorable association.
Kreipe and Mou [28]	2000	Along with techniques for communicating with patients in an efficient manner, practical answers to everyday issues are highlighted.
Golden [29]	2003	Adolescents who suffer from eating disorders are likely to experience considerable medical and psychological morbidity.
The McKnight Investigators [30]	2003	Social pressure and an obsession with thin bodies are significant risk factors for the emergence of eating disorders in teenagers.
Coelho et al. [31]	2014	Female athletes are often plagued by eating disorders, which are significant mental illnesses.
Voelker et al. [32]	2015	The importance of adolescent growth in forming body image, the connection between body image and teenage weight, and the fallout from developing a poor body image in adolescence.
Neumark-Sztainer [33]	2009	Here are five evidence-based suggestions that medical professionals may use in clinical, educational, or other contexts to help avoid eating disorders and obesity in teenagers.
Rosen [34]	2010	This study describes the spectrum of treatment options available, including medication. Pediatricians are urged to support laws and regulations that guarantee patients with eating disorders receive the proper medical attention, dietary intervention, mental health therapy, and care coordination.
Robin et al. [35]	1998	Practitioners are advised to customize these therapies for their children and adolescents with eating disorders by using a stepped-care, decision-tree model of intervention that considers the interventions' cost, intrusiveness, and efficacy.

## Conclusions

Internet usage has been linked to concerns related to food and body image, with teenagers being particularly vulnerable. Social media platforms that highlight physical attractiveness contribute to heightened levels of stress and eating disorders. It is crucial for researchers and professionals to explore how the Internet can empower young adolescents and foster a positive digital environment. Online activism can advocate for societal changes and redirect the focus from unrealistic beauty standards. The Internet and SNS will continue to be significant influencers for body image and eating disorders. Adolescents represent a crucial demographic for examining the negative consequences of Internet usage on body image and eating habits. Constructing evidence-based models can guide preventive measures and interventions.

## References

[1] Saul JS, Rodgers RF (2018). Adolescent eating disorder risk and the online world. Child Adolesc Psychiatr Clin N Am.

[2] Chisuwa N, O'Dea JA (2010). Body image and eating disorders amongst Japanese adolescents. A review of the literature. Appetite.

[3] Holland G, Tiggemann M (2016). A systematic review of the impact of the use of social networking sites on body image and disordered eating outcomes. Body Image.

[4] Prieler M, Choi J (2014). Broadening the scope of social media effect research on body image concerns. Sex Roles.

[5] Fardouly J, Diedrichs PC, Vartanian LR, Halliwell E (2015). Social comparisons on social media: the impact of Facebook on young women's body image concerns and mood. Body Image.

[6] Singh MM, Parsekar SS, Bhumika TV (2016). Body image, eating disorders and role of media among Indian adolescents. J Indian Assoc Child Adolesc Ment Health.

[7] Keery H, van den Berg P, Thompson JK (2004). An evaluation of the Tripartite Influence Model of body dissatisfaction and eating disturbance with adolescent girls. Body Image.

[8] Rodgers RF, Melioli T (2016). The relationship between body image concerns, eating disorders and internet use, part I: a review of empirical support. Adolesc Res Rev.

[9] Franchina V, Coco GL (2018). The influence of social media use on body image concerns. Int J Psychoanal Educ.

[10] Treasure J, Duarte TA, Schmidt U (2020). Eating disorders. Lancet.

[11] (2023). Eating disorders. https://www.mayoclinic.org/diseases-conditions/eating-disorders/symptoms-causes/syc-20353603.

[12] Phillips KA, Didie ER, Menard W, Pagano ME, Fay C, Weisberg RB (2006). Clinical features of body dysmorphic disorder in adolescents and adults. Psychiatry Res.

[13] Angelakis I, Gooding PA, Panagioti M (2016). Suicidality in body dysmorphic disorder (BDD): a systematic review with meta-analysis. Clin Psychol Rev.

[14] McGuire FH, Goldbach JT, Senese JG, Cabrera JR, Schrager SM, Duncan AE (2023). Longitudinal association of homonegative school climate with body dysmorphic disorder among cisgender sexual minority adolescents: testing mediation through proximal minority stressors. Body Image.

[15] Henn AT, Taube CO, Vocks S, Hartmann AS (2019). Body image as well as eating disorder and body dysmorphic disorder symptoms in heterosexual, homosexual, and bisexual women. Front Psychiatry.

[16] Harrison K, Hefner V (2008). Media, body image, and eating disorders. The handbook of children, media, and development.

[17] Ioannidis K, Taylor C, Holt L (2021). Problematic usage of the internet and eating disorder and related psychopathology: a multifaceted, systematic review and meta-analysis. Neurosci Biobehav Rev.

[18] Susanti RD, Eriyani T (2022). Relationship between the intensity of Instagram utilisation and the tendency of body dysmorphic disorder in female students. Malaysian J Med Health Sci.

[19] Harriger JA, Evans JA, Thompson JK, Tylka TL (2022). The dangers of the rabbit hole: reflections on social media as a portal into a distorted world of edited bodies and eating disorder risk and the role of algorithms. Body Image.

[20] Vall-Roqué H, Andrés A, Saldaña C (2021). The impact of COVID-19 lockdown on social network sites use, body image disturbances and self-esteem among adolescent and young women. Prog Neuropsychopharmacol Biol Psychiatry.

[21] Kaewpradub N, Kiatrungrit K, Hongsanguansri S, Pavasuthipaisit C (2017). Association among Internet usage, body image and eating behaviors of secondary school students. Shanghai Arch Psychiatry.

[22] Frieiro P, González-Rodríguez R, Domínguez-Alonso J (2022). Self-esteem and socialisation in social networks as determinants in adolescents' eating disorders. Health Soc Care Community.

[23] Blowers LC, Loxton NJ, Grady-Flesser M, Occhipinti S, Dawe S (2003). The relationship between sociocultural pressure to be thin and body dissatisfaction in preadolescent girls. Eat Behav.

[24] Croll J (2005). Body image and adolescents. Guidelines for adolescent nutrition services.

[25] Hutchinson DM, Rapee RM (2007). Do friends share similar body image and eating problems? The role of social networks and peer influences in early adolescence. Behav Res Ther.

[26] Lonergan AR, Mitchison D, Bussey K, Fardouly J (2021). Social media and eating and body image concerns among men and boys. Eating disorders in boys and men.

[27] Bavikatty A (2023). Tiktok, body image, and eating behavior: An analysis of college-age women. https://deepblue.lib.umich.edu/handle/2027.42/174735.

[28] Kreipe RE, Mou SM (2000). Eating disorders in adolescents and young adults. Obstet Gynecol Clin North Am.

[29] Golden NH (2003). Eating disorders in adolescence and their sequelae. Best Pract Res Clin Obstet Gynaecol.

[30] (2003). Risk factors for the onset of eating disorders in adolescent girls: results of the McKnight longitudinal risk factor study. Am J Psychiatry.

[31] Coelho GM, Gomes AI, Ribeiro BG, Soares Ede A (2014). Prevention of eating disorders in female athletes. Open Access J Sports Med.

[32] Voelker DK, Reel JJ, Greenleaf C (2015). Weight status and body image perceptions in adolescents: current perspectives. Adolesc Health Med Ther.

[33] Neumark-Sztainer D (2009). Preventing obesity and eating disorders in adolescents: what can health care providers do?. J Adolesc Health.

[34] Rosen DS (2010). Identification and management of eating disorders in children and adolescents. Pediatrics.

[35] Robin AL, Gilroy M, Dennis AB (1998). Treatment of eating disorders in children and adolescents. Clin Psychol Rev.

